# Iowa Farmer’s Advocate

**Published:** 1847-12

**Authors:** 


### THE IOWA FARMERS' ADVOCATE.

We are in the receipt of this spirited monthly, from Burlington, and hope it receives, as it deserves, an extensive patronage. It costs $1 per annum, and the farmer will find it to be a very profitable investment. E.

